# Type 1 autoimmune pancreatitis (IgG4-related disease) presenting as new-onset diabetes mellitus: a case report

**DOI:** 10.1186/s13256-026-06131-7

**Published:** 2026-05-30

**Authors:** Yasmin Trinh, Santosh Chaubey

**Affiliations:** Cairns Hospital, Cairns, Australia

**Keywords:** IgG4-related disease, Type 1 autoimmune pancreatitis, Type 3c diabetes mellitus

## Abstract

**Background:**

Type 1 autoimmune pancreatitis (AIP) is the pancreatic manifestation of IgG4-related disease (IgG4-RD) and is an uncommon cause of diabetes mellitus.

**Case presentation:**

Here, we report the case of a 65-year-old white Australian man who was diagnosed with diabetes mellitus secondary to possible IgG4-related disease and had a good clinical and radiological response to corticosteroid therapy.

**Conclusions:**

This case report highlights that IgG4-related disease should be considered in patients who present with acute hyperglycemia, have negative GAD and IA2 antibody status and lack features of insulin resistance. Furthermore, corticosteroid therapy can lead to an improvement in glycemic control in patients with diabetes mellitus secondary to IgG4-related disease.

**Supplementary Information:**

The online version contains supplementary material available at 10.1186/s13256-026-06131-7.

## Background

IgG4-RD is a chronic, immune-mediated disorder that manifests with multiorgan involvement and fibroinflammatory lesions most often affecting the pancreas, biliary system, lacrimal glands, orbital tissues, salivary glands, lymph nodes, lungs, kidneys, aorta, meninges and thyroid gland [1]. The majority of epidemiological data has come from Japan, and in a nationwide survey conducted in 2009, the incidence of IgG4-RD was 0.28–1.08 per 100,000 people. It affects patients of middle to upper age, with an onset at 50–70 years [2]. The pathological hallmark is dense lymphoplasmacytic infiltrate with IgG4-positive plasma cells, storiform fibrosis and obliterative phlebitis. An elevated serum level of IgG4 is frequent [3]. It has a wide range of manifestations that vary by which organs are involved and that are unified by their histopathologic findings and response to treatment. Pancreatic involvement can result in Type 1 autoimmune pancreatitis (AIP), which can manifest as smoldering and insidious chronic pancreatitis with pancreatic atrophy. This can lead to exocrine pancreatic insufficiency and/or Type 3c diabetes mellitus [4]. The diagnosis is complex and can be challenging as symptoms are often non-specific and can vary depending on which organs are affected. It typically requires histopathological diagnosis to be a definitive diagnosis [3].

We report a case of a 65-year-old man who was referred to the diabetes outpatient service with newly diagnosed insulin requiring diabetes mellitus with severe hyperglycemia and negative anti-GAD, IA2 and Zn-T8 autoantibodies, with a pancreatic lesion noted on CT imaging of the pancreas. Further work up demonstrated significantly elevated IgG4 serum levels.

## Case presentation

A 65-year-old white Australian man was referred to the diabetes outpatient service with a new diagnosis of diabetes mellitus, with a significant deterioration in HbA1c from 5.9 to 16.3% in a short span of 7 months. He had been generally unwell for 6 months with nausea, polyuria, polydipsia and 10 kg of unintentional weight loss. He denied any abdominal pain, jaundice or steatorrhea. He was commenced on subcutaneous insulin glargine by his general practitioner and was taking 36 units once daily for 1 month at the time of review with the endocrinology team.

His past medical history was significant for late-onset primary hypogonadism on regular intramuscular testosterone undecanoate 1000 mg 3 monthly injections. He also had hypertension on Perindopril/Amlodipine 5 mg/5 mg daily, and lower urinary tract symptoms for which he recently commenced on Dutasteride/Tamsulosin 500 mcg/400 mcg daily. He was otherwise a generally fit and well farmer who lived independently on an acreage with his wife. He was an active smoker of 5–10 cigarettes daily and had previous heavy alcohol intake, however ceased 35 years ago.

On examination, he had a mesomorphic build with a weight of 84 kg, height 179 cm and body mass index of 26.2 kg/m^2^. His abdomen was soft and non-tender, with no palpable masses. There were no clinical features of hypercortisolism or insulin resistance.

His relevant investigations are as shown in Table 1.

**Table 1:** Baseline investigations

Parameters	Results (units)	Reference range
Na+	131 (mmol/L)	137–147
K+	4.4 (mmol/L)	3.5–5.0
Creatinine	67 (µmol/L)	60–140
eGFR	> 90 (mL/min/1.73 m^2^)	> 59
HCO3-	24 (mmol/L)	25–33
HbA1c	16.3%	< 5.7%
Anti-GAD Ab	5.1 (IU/mL)	< 8.3
Anti-IA2 Ab	2.5 (IU/mL)	< 10.7
Anti-ZnT8 Ab	< 10 (RU/ml)	< 10
C-peptide (random)	0.3 (nmol/L)	< 2.7
Random blood glucose	21.4 (mmol/L)	3.0–7.7
Triglycerides	9.3 (mmol/L)	< 2.0
Total cholesterol	4.7 (mmol/L)	< 4.0
Eosinophils	0.18 × 10^9^ (/L)	0.04–0.40
IgG4	11.90 (g/L) (1190 mg/dL)	0.03–2.01

There was no fasting c-peptide performed; however, the random c-peptide was low for the degree of hyperglycemia.

**Fig. 1:**
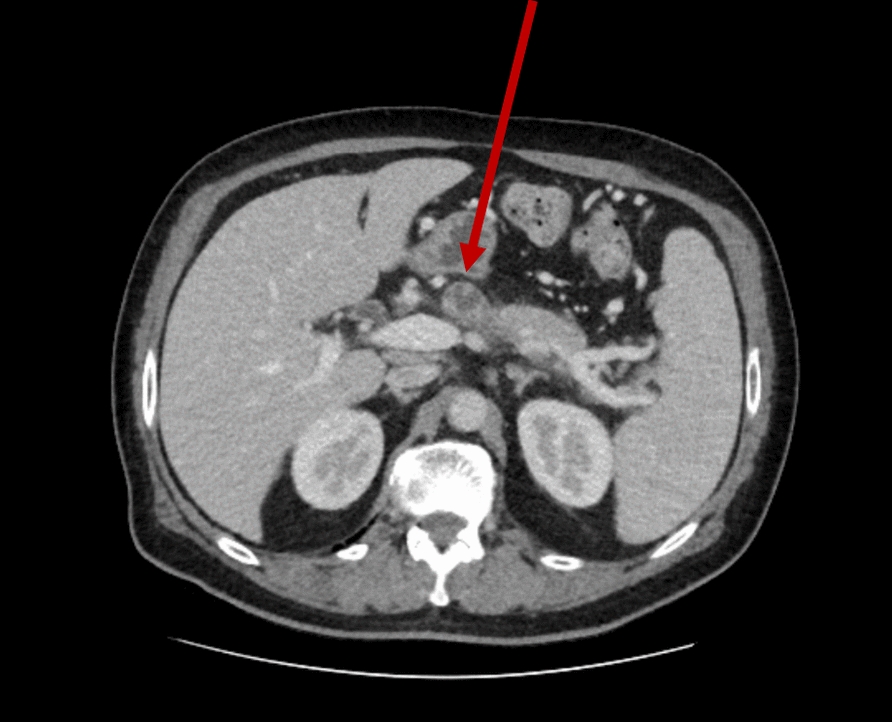
Contrast CT scan of the abdomen. Heterogeneously peripherally enhancing multilobulated structure is abutting the superior margin of the body of the pancreas, 3.4 × 1.8 × 2.0 cm. Unclear if arising from pancreas or a necrotic lymph node. Suspected partial thrombosis of splenic vein and associated splenomegaly

In view of the rapidity of his symptom onset and significant HbA1c rise of 10.4% over a short period of time and negative autoantibody status, a contrast CT scan of the abdomen was performed (Fig. 1). It demonstrated a heterogeneously peripherally enhancing multilobulated structure abutting the superior margin of the body of the pancreas, of size 3.4 × 1.8 × 2.0 cm, but it was unclear if it was arising from the pancreas or reflected necrotic lymph node. There was also a suspected partial thrombosis of the splenic vein and associated splenomegaly. A renal tract ultrasound was also performed due to lower urinary tract symptoms, and it demonstrated a moderately to markedly enlarged prostate gland and an indeterminate nodular structure protruding from the superficial surface of the prostate gland into the lumen of the urinary bladder.

Furthermore, tumor markers demonstrated one elevated CA-19.9 level of 52 U/mL (RI < 30 U/mL), which subsequently normalized on repeat testing to 13 U/mL, and normal CA-125 and CEA levels.

In the context of new-onset insulin-dependent diabetes, negative autoantibodies and a new pancreatic lesion, the patient was diagnosed with Type 3c diabetes mellitus. In view of significant hyperglycemia and osmotic symptoms, he was continued on a basal bolus insulin regimen with insulin glargine injection once daily and insulin aspart injections before main meals.

**Fig. 2:**
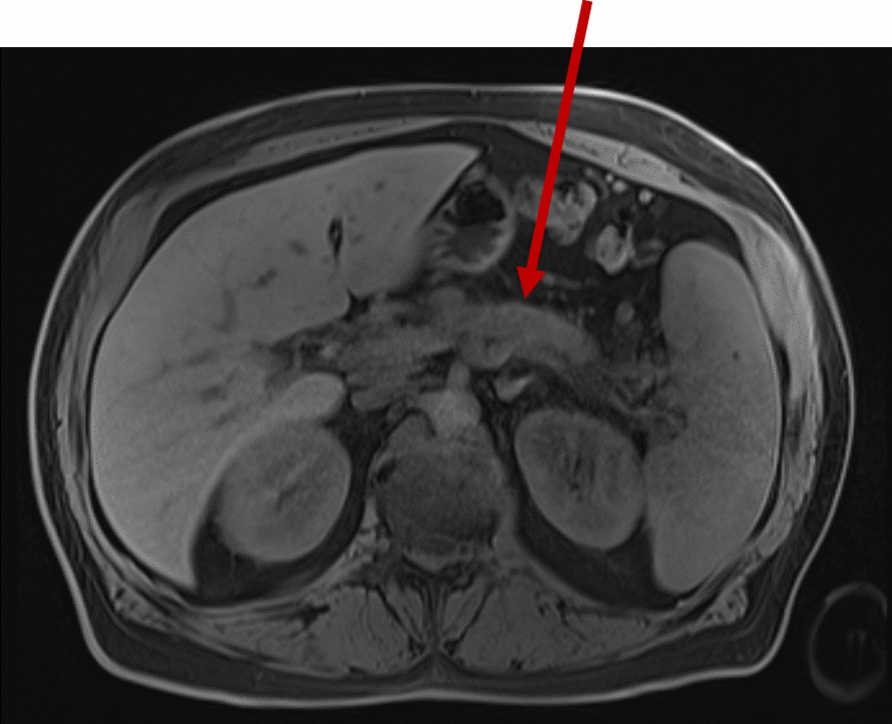
MRI pancreas, T1-weighted view. The pancreas had non-specific changes, including loss of normal lobular pancreatic structure, and the pancreas was diffusely less hyperintense on T1 imaging

**Fig. 3:**
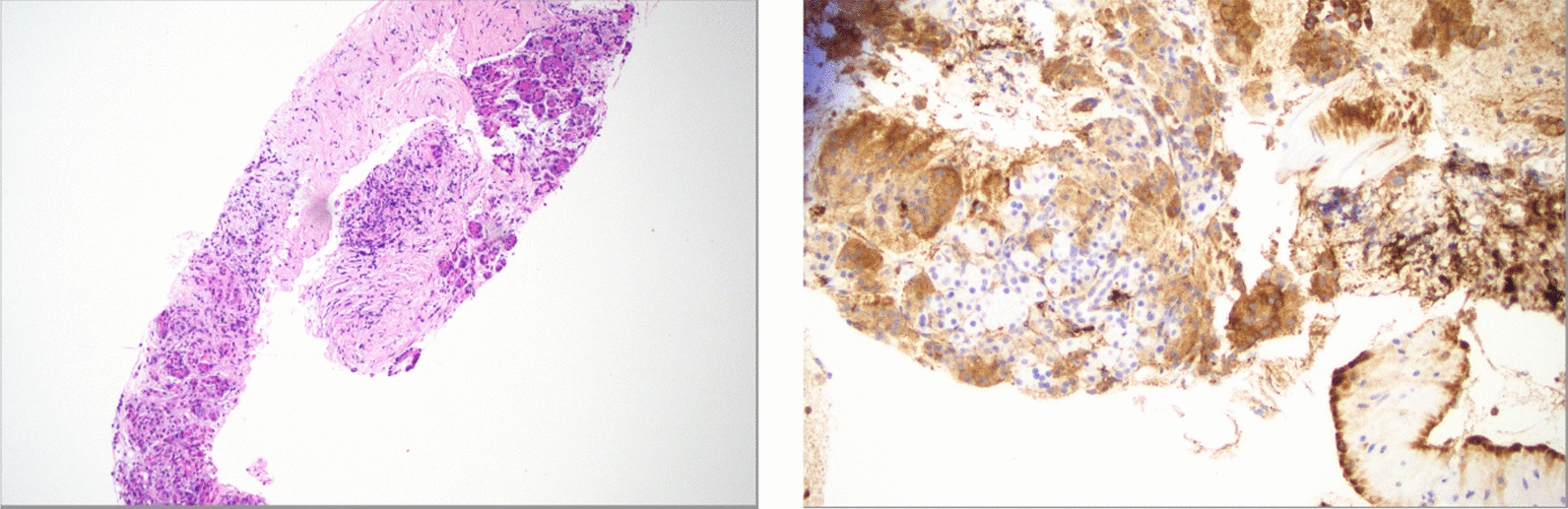
(Left): endoscopic ultrasound biopsy of head of pancreas. Non-specific changes of fibrosis and lymphocytic infiltration. (Right): endoscopic ultrasound biopsy of head of pancreas. IgG4 staining—patchy non-specific staining

Further investigations included a MRI pancreas performed 2 months after the initial contrast CT abdomen (Fig. 2), which demonstrated that the pancreas was not enlarged and there was no focal mass. It had non-specific changes, including loss of normal lobular pancreatic structure and the pancreas was diffusely less hyperintense on T1 imaging, favoring inflammation. There was the presence of small varices around the spleen, related to previously reported partial splenic vein thrombosis. On review of the CT and MRI imaging, no renal or peritoneal involvement was visualized. Subsequently, two endoscopic ultrasounds were performed, which showed a mass of 15 mm × 15 mm in the head of pancreas. Multiple cores and fragments were obtained from the biopsies, of which showed non-specific changes of fibrosis and lymphocytic infiltration; however, no plasma cells, storiform fibrosis or phlebitis was noted (Fig. 3 left). Staining for IgG4 showed patchy non-specific staining, as opposed to staining of plasma cells (Fig. 3 right). IgG4 plasma cells per HPF and IgG4/IgG ratios were not assessed on the biopsies.

The patient had a significant improvement in glycemic control with an improvement in HbA1c from 16.3 to 7.4% within 4 months of commencing insulin treatment. He regained 4 kg of weight during this time. His triglycerides also significantly improved from 9.3 to 2.4 mmol/L. His case was discussed in a multidisciplinary team meeting and the diagnosis of autoimmune pancreatitis type 1 (IgG4-related disease) was considered based on significantly elevated IgG4 level, imaging findings and non-specific histology findings.

**Fig. 4:**
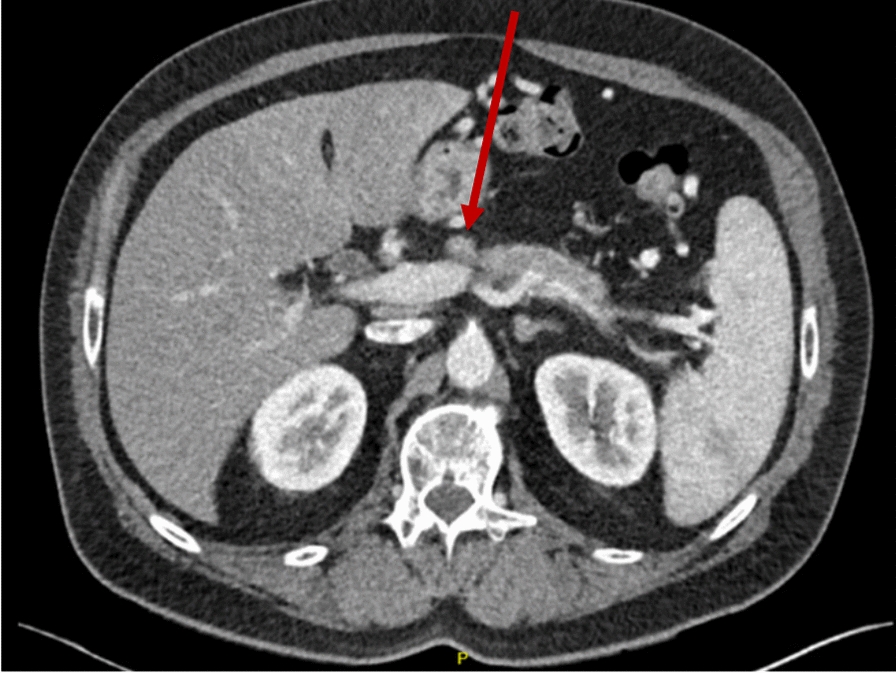
Contrast CT abdomen 3 weeks after commencement of corticosteroids. Solitary fleck of calcification seen in the region of the neck of pancreas, and the previous structure that been at the superior margin of the pancreas was significantly decreased in size

Six months following his initial diagnosis of diabetes, he was commenced on oral Prednisolone 40 mg daily for 4 weeks, followed by a 5 mg/week steroid wean. He gained a further 4 kg of weight within 3 months of starting Prednisolone therapy. A repeat CT abdomen and pelvis (Fig. 4) 3 weeks after commencement of Prednisolone demonstrated a solitary fleck of calcification seen in the region of the neck of pancreas, and the previous structure that been at the superior margin of the pancreas was significantly decreased in size. These radiological improvements in the appearance of the pancreas are suggestive of disease response, which is a hallmark of IgG4-related disease.

The patient was initially uncontactable for 3 months after commencing corticosteroids. He was subsequently reviewed in the diabetes clinic 11 weeks after commencing corticosteroids, at which stage he was on oral Prednisolone 5 mg daily and remained on insulin glargine 25 units daily. A fecal elastase performed 3 months after starting glucocorticoids was 70 µg/g, consistent with severe pancreatic insufficiency, and he was commenced on Creon 50,000–75,000 units with main meals and 25,000 units with snacks. His repeat IgG4 level was 4.12 g/L (412 mg/dL), which was significantly reduced from 11.6 g/L (1190 mg/dL) prior to commencement of oral Prednisolone. He completed an 11-week course of corticosteroids as planned. A FDG-PET was arranged 1 month after cessation of corticosteroids, which demonstrated resolved autoimmune pancreatitis, with no active systemic related IgG4 disease.

His latest HbA1c has improved further to 6.5%, and he has ongoing regular follow-up with the endocrinology and gastroenterology teams.

### Timeline

**Table:** 

T = 0 (April 2023)	Symptom onset HbA1c 5.9%
T = 7 months	HbA1c 16.3% Initial CT abdomen IgG4 level 11.90 g/L (1190 mg/dL)
T = 9 months	HbA1c 7.4% MRI pancreas
T = 12 months	Glucocorticoids commenced
T = 13 months	Follow-up CT pancreas after commencement of glucocorticoids
T = 15 months	Completion of glucocorticoid course Repeat IgG4 level 4.12 g/L (412 mg/dL)
T = 16 months	FDG-PET

## Discussion

Our index patient was diagnosed with new onset diabetes at the age of 65 years. Considering the etiologies of diabetes in this age group, Type 1, Type 2 and secondary diabetes were all considered. In the context of negative autoimmune diabetes antibodies and absence of features of ketoacidosis, a diagnosis of type 1 diabetes was deemed unlikely. T2DM was considered unlikely based on the lack of features of insulin resistance, a very rapid transition from prediabetes to florid, symptomatic insulin-dependent diabetes and a dramatic response to low-dose insulin. Therefore, the high probability of secondary diabetes was considered. He did not have any features of acute pancreatitis, although noting that a lipase was not performed, which is an important marker in assessing for acute pancreatitis. He did have a distant history of heavy alcohol intake, however, was now abstinent and there were no symptoms to suggest chronic pancreatitis. With a significantly elevated IgG4 level over five times the upper limit of normal on two occasions and pancreatic abnormalities demonstrated on imaging, Type 3c diabetes mellitus secondary to IgG4-related disease was suspected. There is no one universal diagnostic criteria for IgG4-related disease. The 2020 Revised Comprehensive Diagnostic Criteria for IgG4-related disease is frequently referred to in the literature (Table 2) [5]. Based on this criteria, which had a sensitivity of 100% in a single center, retrospective study [6], this patient’s case would be considered a possible diagnosis of IgG4-related disease, with elevated serum IgG4 levels and radiological abnormality of localized swelling or mass in an organ.

**Table 2:** 2020 revised comprehensive diagnostic (RCD) criteria for IgG4-RD

1. Clinical and radiological features • One or more organs show diffuse or localized swelling or a mass or nodule characteristic of IgG4-RD. In single organ involvement, lymph node swelling is omitted
2. Serological diagnosis • Serum IgG4 levels greater than 135 mg/dL
3. Pathological diagnosis Positivity for two of the following three criteria: • Dense lymphocyte and plasma cell infiltration with fibrosis • Ratio of IgG4-positive plasma cells/IgG-positive cells greater than 40% and the number of IgG4-positive plasma cells greater than 10 per high powered field • Typical tissue fibrosis, particularly storiform fibrosis, or obliterative phlebitis
Diagnosis: • Definite: (1) + (2) + (3) • Probable: (1) + (3) • Possible: (1) +( 2)

Interestingly, this patient’s histopathological features were not typical for IgG4-related disease; however, serial imaging 3 weeks after commencement of corticosteroids demonstrated an improvement in the appearance, and the previous structure initially seen at the superior margin of the pancreatic head had significantly decreased in size, suggestive of disease response. Furthermore, a repeat FDG-PET 1 month after completion of the oral Prednisolone course demonstrated resolved autoimmune pancreatitis. A prompt improvement after starting corticosteroid treatment is often interpreted as a useful diagnostic sign for autoimmune pancreatitis in patients with an unclear diagnosis.

Corticosteroids are the mainstay of treatment for AIP. There are several corticosteroid dosage recommendations, including Prednisolone 0.4–0.6 mg/kg/day for 2–4 weeks, reduced by 5 mg/day every 1–2 weeks until 10–15 mg/day, and then gradually tapered to a maintenance dose of 2.5–5 mg/day over a period of 2–3 months [7]. Another regimen is Prednisolone 40 mg/day for 4 weeks, followed by a 7-week taper with a dose reduction of 5 mg per week to cessation (11 week duration of treatment) [8]. Current guidelines recommend low-dose (5 mg/day) maintenance corticosteroid treatment for 2–3 years to reduce the risk of disease relapse [7].

Several studies have explored the impact of corticosteroid therapy in AIP on glycemic control in patients with diabetes. A retrospective study of 69 patients with AIP found that at 3 months after commencing corticosteroid therapy, improved glucose tolerance (defined as an improvement in HbA1c by at least > 0.5%) was seen in 54% of all diabetes mellitus patients [9].

With regards to the underlying mechanism of change in glycemic control, a retrospective study of 61 patients with Type 1 AIP found that at 4 weeks after commencing corticosteroid therapy, C-peptide index significantly increased in patients who were diagnosed with concurrent diabetes at time of their AIP diagnosis, whereas the C-peptide index tended to decrease in those with pre-existing diabetes [10]. At 1 and 2 years after commencement of corticosteroid treatment, the prevalence of diabetes in those with concurrent diabetes diagnosis decreased from 100 to 77%, whereas the prevalence of diabetes in those with pre-existing diabetes did not change significantly. This particular study demonstrated that perhaps it is the improved insulin secretion from corticosteroid therapy that allows for long-term improvement of diabetes. It also suggested that having concurrent diabetes with the Type 1 AIP diagnosis has improved glycemic outcomes compared to those who had pre-existing diabetes.

## Discussion and conclusions

Autoimmune pancreatitis secondary to IgG4-RD is an uncommon condition. It is a multisystem disease with a wide range of clinical presentations. It is often an underdiagnosed, misdiagnosed and underreported disease, which can have long-term consequences even apart from those related to diabetes mellitus. IgG4-related disease should be considered in patients who present with acute hyperglycemia, have negative GAD and IA2 antibody status and lack features of insulin resistance. A good response to oral corticosteroids is a unique feature of this condition whereby corticosteroids counterintuitively assist in achieving improved glycemic control in those with a concurrent diagnosis of diabetes mellitus at time of AIP diagnosis.

## Supplementary Information

Additional file1.

## Data Availability

Data sharing is not applicable to this article as no datasets were generated or analyzed during the current study.

## Abbreviations

AIP: Autoimmune pancreatitis
IgG4-RD: IgG4-related disease
