# Supplementary material for: Type 1 autoimmune pancreatitis (IgG4-related disease) presenting as new-onset diabetes mellitus: a case report
Source: J Med Case Rep. 2026 May 30;20:438. doi: 10.1186/s13256-026-06131-7 (PMC13430874; doi:10.1186/s13256-026-06131-7)
Supplement: Supplementary file 1 — Additional file1. [file 13256_2026_6131_MOESM1_ESM.pdf]

2013 CARE Checklist

1. **Title**
    - Type 1 Autoimmune Pancreatitis (IgG4-related disease) presenting as new onset diabetes mellitus: a case report.
2. **Key Words**
    - IgG4-related disease, diabetes mellitus, case report.
3. **Abstract**
    - **Background** Type 1 Autoimmune Pancreatitis (AIP) is the pancreatic manifestation of IgG4-related disease (IgG4-RD) and is an uncommon cause of Diabetes Mellitus.
    - **Case Presentation** Here, we report the case of a 65-year-old Caucasian man who was diagnosed with Diabetes Mellitus secondary to IgG4-related disease and had a good clinical and radiological response to corticosteroid therapy.
    - **Conclusions** This case report highlights that IgG4-related disease should be considered in patients who present with acute hyperglycaemia, have negative GAD and IA2 antibody status and lack features of insulin resistance. Furthermore, corticosteroid therapy can lead to an improvement in glycaemic control in patients with Diabetes Mellitus secondary to IgG4-related disease.
4. **Introduction**
    - This case demonstrates improvement in glycaemic control in patients with Diabetes Mellitus secondary to IgG4-related disease, which is a unique and uncommon condition.
5. **Patient Information**
    - 65 year old male, Caucasian.
    - Past medical history:
        1. Primary hypogonadism.
        2. Hypertension.
        3. Lower urinary tract symptoms on Dutasteride/Tamsulosin.
    - Medications:
        1. Testosterone undeconate 1000mg 3 monthly injections.
        2. Perindopril/Amlodipine 5mg/5mg daily.
        3. Dutasteride/Tamsulosin 500mcg/400mcg daily.
    - Psychosocial history:
        1. Works as a farmer, lives on an acreage with wife.
        2. Independent with all activities of daily living.
        3. Active smoker – 5-10 cigarettes daily.

4. Previous heavy alcohol intake, ceased 35 years ago.

6. **Clinical Findings**
   - Weight 84kg, height 179cm, BMI 26.2 kg/m^2.
   - Abdomen soft and non-tender, no palpable masses.
   - No clinical features of hypercortisolism or insulin resistance.

7. **Timeline** – Historical and current information from this episode of care organized as a timeline (figure or table).

Reviewed in Diabetes outpatient clinic as a new case, with a new diagnosis of Diabetes Mellitus. Had been commenced on insulin glargine 36 units once daily by his General Practitioner one month prior to the Diabetes Clinic appointment
Initial investigations (biochemistry) were completed.
CT abdomen performed.
MRI abdomen performed 2 months after initial CT abdomen.
Two endoscopic ultrasounds (and biopsy of head of pancreas lesion) performed due to MRI findings.
Commenced on Prednisolone 40mg daily for 4 weeks (commenced six months following initial diagnosis of diabetes).
Repeat CT abdomen/pelvis 3 weeks after commencement of Prednisolone demonstrated radiological improvement in appearance of the pancreas, suggestive of disease response.
Prednisolone wean 5mg/week, with regular follow-up in Gastroenterology clinic.
Patient was uncontactable for three months after starting corticosteroids.
Subsequently returned to follow-up with Endocrinology and Gastroenterology teams. FDG PET performed one month after cessation of corticosteroids demonstrated resolved autoimmune pancreatitis.

8. **Diagnostic Assessment**
   - **Table 1: Baseline investigations**

Parameters	Results (units)	Reference range
**Random blood glucose**	21.4 (mmol/L)	3.0-7.7
**Na+**	131 (mmol/L)	137-147
**K+**	4.4 (mmol/L)	3.5-5.0
**Creatinine**	67 (umol/L)	60-140
**eGFR**	>90 (ml/min/1.73m^2)	>59
**HCO3-**	24 (mmol/L)	25-33
**HbA1c**	16.3%	<5.7%
**Anti GAD Ab**	5.1 (IU/mL)	<8.3
**Anti IA2 Ab**	2.5 (IU/mL)	<10.7
**Anti ZnT8 Ab**	<10 (RU/ml)	<10
**C-peptide (random)**	0.3 (nmol/L)	
**Triglycerides**	9.3 (mmol/L)	<2.0
**Total cholesterol**	4.7 (mmol/L)	<4.0
**IgG4**	11.90 (g/L)	0.03-2.01

- CA 19.9 52U/ml (RI <30U/ml), repeat CA 19.9 13U/ml
- CT abdomen with contrast: heterogeneously peripherally enhancing multilobulated structure abutting the superior margin of the body of the pancreas, of size 3.4x1.8x2.0cm, but it was unclear if it was arising from the pancreas or reflected necrotic lymph node. There was also a suspected partial thrombosis of the splenic vein and associated splenomegaly.
- Renal tract ultrasound: moderately to markedly enlarged prostate gland and an indeterminate nodular structure protruding from the superficial surface of the prostate gland into the lumen of the urinary bladder.
- MRI pancreas: pancreas not enlarged, no focal mass. Non-specific changes including loss of normal lobular pancreatic structure and the pancreas was diffusely less hyperintense on T1 imaging, favouring inflammation. There was the presence of small varices around the spleen, related to previously reported partial splenic vein thrombosis.
- Two endoscopic ultrasounds: demonstrated a mass of 15 mmx 15 mm in the head of pancreas, biopsies of which showed non-specific changes of fibrosis and lymphocytic infiltration, however no plasma cells, storiform fibrosis or phlebitis was noted (Fig. 3). Staining for IgG4 showed patchy non-specific staining, as opposed to staining of plasma cells (Fig. 4).
- Diagnosis: Autoimmune Pancreatitis Type 1 (IgG4-related disease). Diagnosis was made based on significantly elevated IgG4 level, imaging findings and non-specific histology findings from endoscopic ultrasound.

9. **Therapeutic Intervention**

   - Commenced on insulin therapy at time of diabetes diagnosis.
   - Commenced on Prednisolone 40mg daily six months following initial diagnosis of diabetes (following diagnosis of Autoimmune Pancreatitis Type 1). Prednisolone 40mg daily for 4 weeks, followed by a 5mg/week steroid wean.

10. **Follow-up and Outcomes**

- The patient had a significant improvement in glycaemic control with an improvement in HbA1c from 16.3% to 7.4% within four months of commencing insulin treatment. He also regained 4kg of weight during this time. Triglyceride levels improved from 9.3mmol/L to 2.4mmol/L with insulin therapy.
- Three weeks after commencement of Prednisolone, a repeat CT abdomen/pelvis demonstrated a solitary fleck of calcification seen in the region of the neck of pancreas, and the previous structure that been at the superior margin of the pancreas was significantly decreased in size. These radiological improvements in the appearance of the pancreas are suggestive of disease response, which is a hallmark of IgG4 related disease.
- The patient was initially uncontactable for three months after commencing corticosteroids. He was subsequently reviewed in the Diabetes Clinic 11 weeks after commencing corticosteroids, at which stage he was on oral Prednisolone 5mg daily and remained on insulin glargine 25 units daily. A faecal elastase was 70 ug/g, consistent with severe pancreatic insufficiency, and he was commenced on Creon 50,000-75,000 units with main meals and 25,000 units with snacks. His repeat IgG4 level was 4.12 g/L, which was significantly reduced from 11.6 g/L prior to commencement of oral Prednisolone.
- The patient completed an 11-week course of corticosteroids as planned. A FDG-PET was arranged one month after cessation of corticosteroids, which demonstrated resolved autoimmune pancreatitis, with no active systemic related IgG4 disease.
- His latest HbA1c has improved further to 6.5%, and he has ongoing regular follow-up with the Endocrinology and Gastroenterology teams.

11. **Discussion**

- The strengths to our approach of this patient's case were:
    1. The patient's case was also discussed in a multidisciplinary team meeting, and the diagnosis of Autoimmune Pancreatitis Type 1 was made at that time.
    2. There was regular follow-up of the patient following commencement of treatment, and parameters including repeat imaging, HbA1c and lipids were arranged to assess response to corticosteroid therapy.
- Corticosteroids are the mainstay of treatment for autoimmune pancreatitis.
- There are several corticosteroid dosage recommendations, including Prednisolone 0.4-0.6mg/kg/day for 2-4 weeks, reduced by 5 mg/d every 1–2 weeks until 10–15 mg/day, and then gradually tapered to a maintenance dose of 2.5–5 mg/day over a period of 2–3 months.[7] Another regimen is Prednisolone 40mg/day for 4 weeks, followed by a 7-week taper with a dose reduction of 5mg per week to cessation (11

week duration of treatment).[8] Current guidelines recommend low-dose (5mg/day) maintenance corticosteroid treatment for 2-3 years to reduce the risk of disease relapse. [7]

- Several studies have explored the impact of corticosteroid therapy in autoimmune pancreatitis on glycaemic control in patients with diabetes. With regards to the underlying mechanism of change in glycaemic control, a retrospective study of 61 patients with Type 1 AIP found that at four weeks after commencing corticosteroid therapy, C-peptide index significantly increased in patients who were diagnosed with concurrent diabetes at time of their AIP diagnosis, whereas the C-peptide index tended to decrease in those with pre-existing diabetes.[10]
- The primary 'take-away' lessons from this case report is that IgG4-related disease should be considered in patients who present with acute hyperglycaemia, have negative autoantibodies and lack of features of insulin resistance, as treatment with corticosteroid therapy in this setting can lead to a significant improvement in glycaemic control.

12. **Informed Consent** – Verbal and written informed consent was obtained from the patient.
